# IFI35 limits antitumor immunity in triple-negative breast cancer via CCL2 secretion

**DOI:** 10.1038/s41388-023-02934-w

**Published:** 2024-01-12

**Authors:** Baojin Xu, Hefen Sun, Simeng Liu, Li Liao, Xiaoqing Song, Yi Wu, Yifeng Hou, Wei Jin

**Affiliations:** 1 Key Laboratory of Breast Cancer in Shanghai, Fudan University Shanghai Cancer Center, Shanghai, 200032 China; 2 Department of Oncology, Shanghai Medical College, Fudan University, Shanghai, 200032 China; 3 Department of Breast Surgery, Liaoning Cancer Hospital and Institute, Cancer Hospital of China Medical University, Shenyang, 110042 China

**Keywords:** Cancer microenvironment, Tumour immunology

## Abstract

Triple-negative breast cancer (TNBC) is the most aggressive subtype of breast cancer with poor prognosis due to the lack of therapeutic targets. Although immunotherapy brings survival benefits to patients diagnosed with TNBC, it remains limited and treatment resistance is widespread. Here we demonstrate that IFI35 is highly expressed in tumor tissues and can be induced by Interferon-γ in a time-dependent and concentration-dependent manner in breast cancer cells. In xenograft models, we reveal that IFI35 dramatically increases myeloid-derived suppressor cells infiltration in tumors, along with depletion and anergy of CD8^+^T cells. IFI35 ablation leads to prolonged survival of the mice. Mechanistically, RNA-sequencing reveals that IFI35 promotes CCL2 secretion, resulting in the remodeling of TNBC immune microenvironment. Ablation of IFI35 promotes the infiltration of effector CD8^+^T cells, and thereby sensitizes TNBC to anti-PD-1 immunotherapy. Our data suggest that IFI35 limits antitumor immunity and may be expected to become a new immunotherapy target in TNBC.

## Introduction

Breast cancer is the most common cancer in women. Triple-negative breast cancer (TNBC) is the most aggressive subtype of breast cancer. It accounts for 10%-15% of all breast cancers, and lacks therapeutic targets due to its lack of expression of ER, PR, and HER2 receptors [1]. In the past, chemotherapy and radiotherapy were the main treatment strategies. The development of immune checkpoint inhibitors (ICIs) has initiated the era of immunotherapy for TNBC [2]. ICIs can provide survival benefits in TNBC, but few patients achieve a durable clinical response [3]. Therefore, identifying new immunotherapeutic targets will bring new hope for TNBC immunotherapy.

Interferons (IFNs) are named for their ability to interfere with viral replication in the host, and contain three major family members [4]. Type I IFNs are mainly expressed by innate immune cells. Type II IFN contains only one member IFN-γ and is induced by activated NK cells and T cells. Type III IFNs are restricted in their tissue distribution. All these secreted cytokines can drive specific gene expression signatures that can be overlapping and crosstalk with each other, causing dynamic and complex cascades that enhance or weaken interferon response. Till now, the theory of cancer immunoediting indicates that IFNs are a double-edged sword [5, 6]. On the one hand, they can promote anti-tumor immune responses to eliminate tumor cells; on the other hand, they can reshape tumor characteristics that contribute to tumor immune evasion. Thus, there remains an unmet need to better elucidate the role of IFNs.

Interferon-γ (IFN-γ) is a pleiotropic cytokine, and is actively involved in the regulation of tumor immunity [7, 8]. Under the stimulation of IFN-γ, IFNGR1 and IFNGR2 activate downstream IFN-stimulated response elements through JAK1/JAK2-STAT1 signaling cascade, causing the expression of a series of IFN-stimulated genes (ISGs) [4]. As one of the classical ISGs, PD-L1 has been confirmed to play an oncogenic role in promoting malignant tumor progression [9]. Also, a series of clinical trials showed that anti-PD-1/PD-L1 immunotherapy inhibits tumor growth and prolongs patient survival [10–13]. Interestingly, the association of PD-L1 expression with patient prognosis remains controversial. Some evidence suggests that PD-L1 is associated with good prognosis [14–17], but other studies have reached the opposite conclusion [18–20]. It can be seen that the relationship between the actual role of ISGs and the prognosis of patients is not always consistent. In our previous study, IFI35 as an ISG was identified to be involved in the M1 polarization of macrophages and patients with high IFI35 expression had better prognosis [21]. It seems that IFI35 is a tumor suppressor, but its actual role in the tumor immune microenvironment (TIME) still remains to be demonstrated in animal models.

Here, for the first time, we explored the role of IFI35 in the TIME of TNBC using IFI35-deficient genetically engineered mice and syngeneic xenograft mouse models. In genetically engineered mouse models proficient or deficient of IFI35, we showed that its deficiency in immune cells does not affect tumor growth. In xenograft models, we found that IFI35 promoted TNBC immune escape in a tumor cell-intrinsic manner. In addition, IFI35 ablation obviously inhibited tumor growth and extended survival. The combination of IFI35 ablation and anti-PD-1 can exert a synergistic therapeutic effect. Therefore, targeting IFI35 may be a promising way to improve the efficacy of ICIs in TNBC.

## Results

### IFN-γ induces IFI35 expression in TNBC

**Fig. 1:**
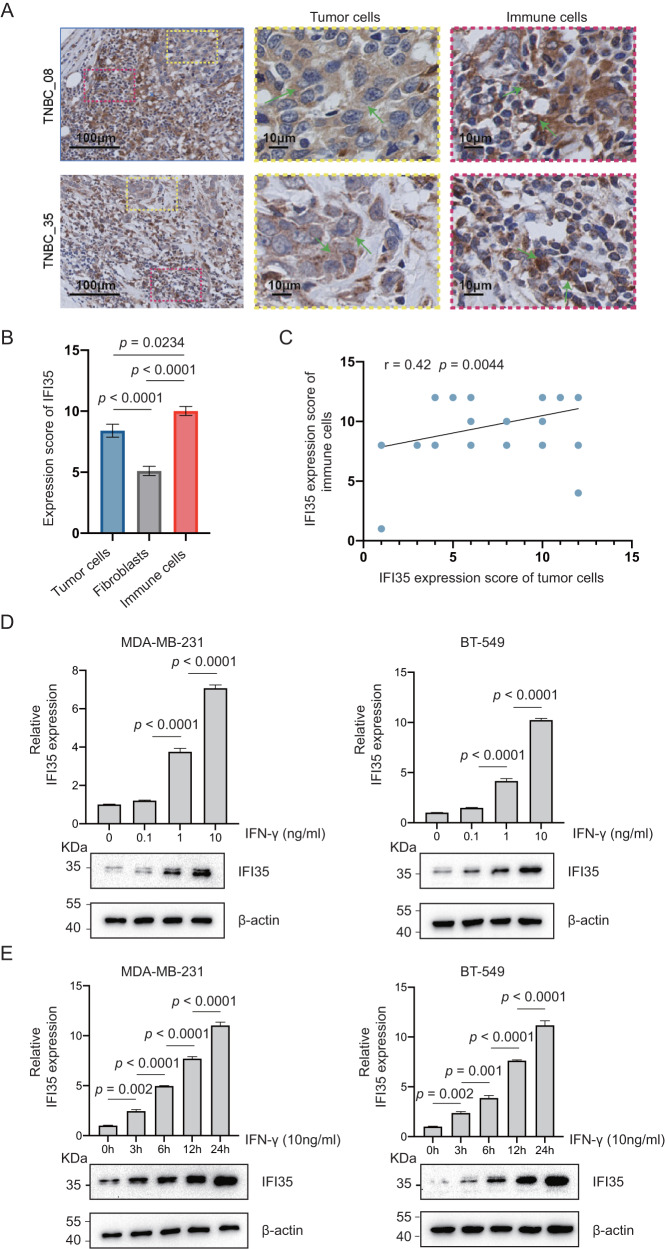
IFN-γ induces IFI35 expression in tumor microenvironment. **A** Representative immunostainings for IFI35 in TNBC patients’ sample (*n* = 45). **B** Histological quantification of IFI35 expression in tumor cells, fibroblasts, immune cells within (**A**) (one-way ANOVA, Tukeys’s multiple comparison test, mean with SEM is plotted). **C** Correlation of IFI35 expression in tumor and immune cells (Pearson, *n* = 45 pairs, two-tailed). **D**, **E** RT-qPCR and Immunoblot analysis of BT-549 or MDA-MB-231 with indicated IFN-γ for IFI35 and β-actin as loading control (one-way ANOVA, Tukeys’s multiple comparison test, mean with SEM is plotted).

Several studies have indicated that IFI35 is expressed in the cytoplasm of various cells including immune cells, epithelial cells and fibroblasts [22]. From the TISCH scRNA-seq database, we found that IFI35 was widespread in various types of cells in breast cancer (Supplementary Fig. S1). To better assess the expression of IFI35 in TNBC, we performed immunohistochemical staining on 45 samples from TNBC patients. As expected, IFI35 was expressed in the cytoplasm of immune cells, tumor cells, and fibroblasts (Fig. 1A). The expression level was highest in immune cells, followed by tumor cells and fibroblasts (Fig. 1B, Supplementary Table 1). In addition, the expression of IFI35 was positively correlated in immune and tumor cells (Fig. 1C). Some studies have noted that IFN-γ can induce the expression of IFI35 in HeLa cervical cancer cells and THP-1 monocytes, as IFI35 is an IFN-inducible protein [22, 23]. Therefore, we used RT-qPCR and western blot to determine whether IFN-γ could induce the expression of IFI35 in TNBC cell lines. Consistent with the previous result [24], we further demonstrated that IFI35 expression was induced by IFN-γ in MDA-MB-231 and BT-549 TNBC cells in a time- and concentration-dependent manner (Fig. 1D, E). Collectively, it shows that IFI35 is highly expressed in TNBC tumor cells in response to IFN-γ stimulation.

### The antitumor immune response is not affected in Ifi35^−/−^ mice

Given that IFI35 was highly expressed in TNBC immune microenvironment, to evaluate the function of IFI35 in the TNBC immune microenvironment, we generated Ifi35^−/−^ mice on a balb/c genetic background by deleting 6 exons of the Ifi35 gene (Supplementary Fig. S2A, B). Ifi35^−/−^ mice had no obvious defects in growth or development, showing normal fertility, breeding, and body weight (data not shown). Analysis of the immune system revealed no significant change in the proportions of NKp46^+^ natural killer (NK) cells, CD4^+^T cells, CD8^+^T cells, CD4^+^CD25^+^ regulatory T (T_reg_) cells, CD11b^+^CD11c^+^ dendritic cells (DCs), CD11b^+^Ly6C^+^ monocytes, CD11b^+^Ly6G^+^ neutrophils, or CD11b^+^F4/80^+^ macrophages in the spleen of Ifi35^−/−^ mice (Supplementary Figs. S2C and S3), suggesting that the mice had no alteration of global immune cell populations at steady state. When challenged with the 4T1 or EMT6 TNBC cell line in the mammary fat pad, no difference in tumor growth was observed in Ifi35^−/−^ mice (Supplementary Figs. S2D, E and S4). Taken together, these results show that Ifi35 deficiency in immune cells does not affect syngeneic tumor growth.

### Tumor-intrinsic IFI35 drives TNBC immune evasion

**Fig. 2:**
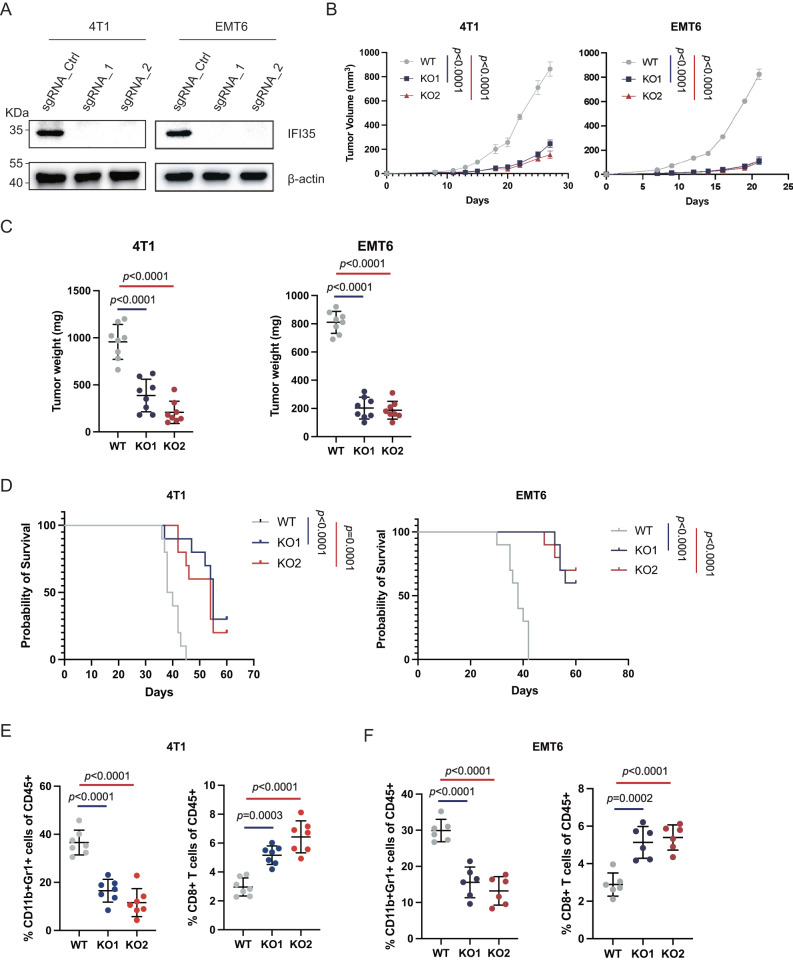
IFI35 promotes TNBC progression via MDSCs recruitment. **A** Immunoblot analysis of IFI35 protein level in 4T1 or EMT6 mouse TNBC cells transduced with sgRNA targeting Ifi35 and control. **B** Tumor growth curves of balb/c mice inoculated mammary fat pad with WT or fi35^ko^ 4T1 and EMT6 tumor cells (two-way ANOVA test, *n* = 8). **C** Tumor weight 30 days after tumor inoculation (*n* = 8, one-way ANOVA test with Turkey’s multiple comparisons, Mean with SD is plotted). **D** Kaplan–Meier curves for the survival of balb/c mice with Ifi35^ko^ or WT 4T1 and EMT6 tumor cells (*n* = 10 per group, log-rank test). The frequencies of MDSCs and CD8^+^T cells population in balb/c mice inoculated with Ifi35^ko^ or WT 4T1 (**E**) and EMT6 (**F**) tumor cells (*n* = 6-7, one-way ANOVA test with Turkey’s multiple comparisons, mean with SD is plotted).

To investigate whether tumor-intrinsic IFI35 affects the antitumor immune response, the Ifi35 gene was knocked out in the 4T1 or EMT6 TNBC mouse cell lines (named as Ifi35^ko^ cells, sgRNA_Ctrl refers to WT, sgRNA_1 refers to KO1, and sgRNA_2 refers to KO2). As shown in Fig. 2A, Ifi35^ko^ cells hardly express the IFI35 gene when compared with WT. In vitro, we observed that Ifi35^ko^ cells had similar viability to control cells (Supplementary Fig. S5A). This result was supported by an analysis of public genome-wide CRISPR–Cas9 screening data [25] (Supplementary Fig. S5B). Interestingly, the tumorigenicity of the Ifi35^ko^ 4T1 or EMT6 cell lines was significantly inhibited in vivo (Fig. 2B). The tumor mass was reduced more than twofold compared with the control group at the study endpoint (Fig. 2C). In addition, mice inoculated with the Ifi35^ko^ 4T1 or EMT6 cell lines had prolong survival (Fig. 2D).

**Fig. 3:**
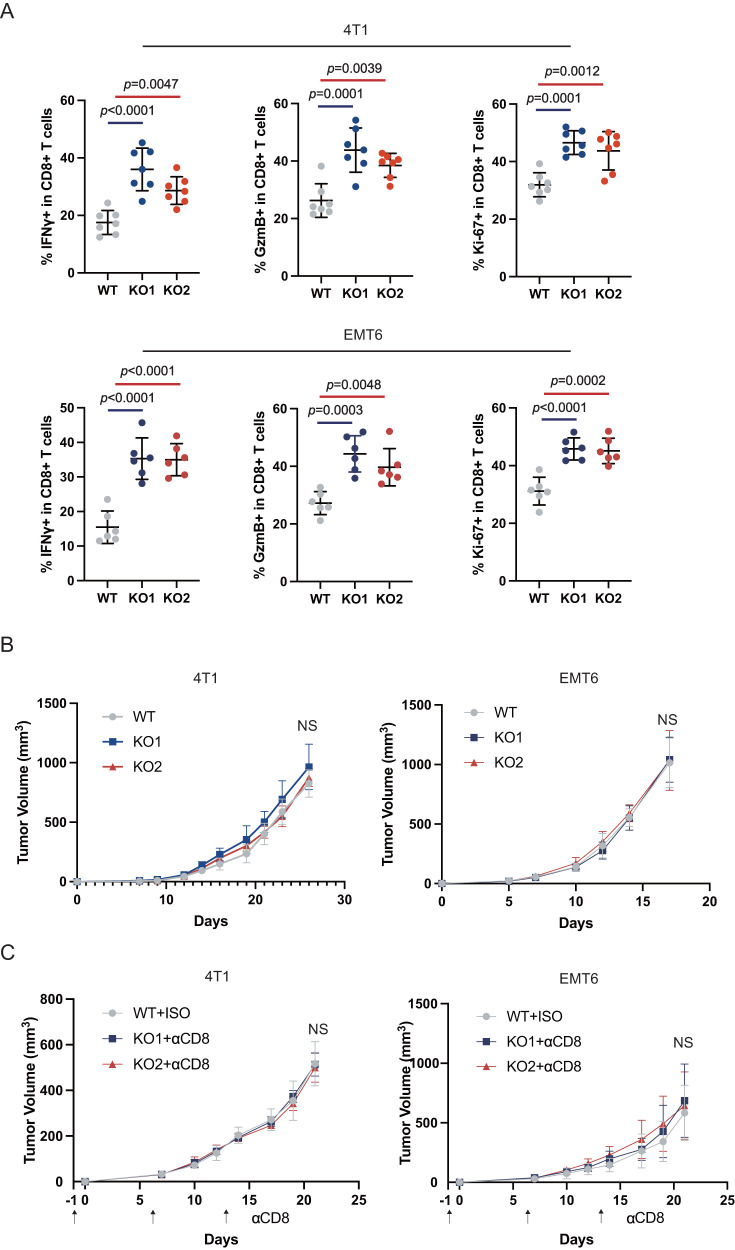
CD8^+^T cells exert anti-tumor effector functions. **A** The frequencies of IFNγ^+^CD8^+^, GzmB^+^CD8^+^ and Ki-67^+^CD8^+^ T cells population in balb/c mice inoculated with Ifi35^ko^ or WT 4T1 and EMT6 tumor cells (*n* = 6-7, one-way ANOVA test with Turkey’s multiple comparisons, mean with SD is plotted). **B** Tumor growth curves of nude mice inoculated mammary fat pad with Ifi35^ko^ or WT 4T1 or EMT6 tumor cells (*n* = 6-8 per group, two-way ANOVA test, mean with SD is plotted). **C** Ifi35^ko^ or WT 4T1 and EMT6 tumor growth curves of Balb/c mice treated with anti-mouse CD8 antibody or isotype control (*n* = 5 per group, two-way ANOVA test, mean with SD is plotted).

To comprehensively characterize the changes in the TIME, we performed flow cytometry analysis of tumor tissues (Supplementary Fig. S6). Compared with wild-type (WT) tumors, Ifi35^ko^ tumors had decreased the frequencies of infiltrating myeloid-derived suppressor cells (MDSCs, CD11b^+^Gr1^+^) (Fig. 2E, F), and M2 macrophages (CD11b^+^F4/80^+^CD206^+^), and increased frequencies of infiltrating DCs (CD11b^+^CD11c^+^) and M1 macrophages (CD11b^+^F4/80^+^CD86^+^) (Supplementary Fig. S7), and the difference in MDSCs was the most obvious. These changes constituted an antitumor immune microenvironment. In addition, the percentage of CD8^+^T cells (Fig. 2E, F), but not CD4^+^T cells or T_reg_ cells (CD4^+^CD25^+^) (Supplementary Fig. S7), was significantly increased in Ifi35^ko^ tumors. The production of IFN-γ and GzmB by CD8^+^T cells within Ifi35^ko^ tumors was enhanced (Fig. 3A), indicating stronger antitumor ability. Additionally, the proportion of Ki-67^+^CD8^+^T cells was increased (Fig. 3A), suggesting an elevated proliferation ability of CD8^+^T cells. However, the proportion of NK (NKp46^+^) cells was similar in the Ifi35^ko^ and WT tumors (Supplementary Fig. S7). The expression of PD-1 can mediate the activation of co-inhibitory signaling in CD8^+^T cells to inhibit their antitumor function. Therefore, we asked whether Ifi35^ko^ tumors had abnormal PD-1 expression on the surface of CD8^+^T cells. The results showed that PD-1 expression was similar in the Ifi35^ko^ and WT 4T1 tumors (Supplementary Fig. S7).

CD8^+^T cells, as the crucial antitumor immune cells participating in cell killing, were significantly increased in Ifi35^ko^ tumors. To investigate whether the improved CD8^+^T cells response mediated the tumor reduction in the Ifi35^ko^ group, we inoculated Ifi35^ko^ and WT 4T1 or EMT6 cells into nude mice (deficient in T cells). As expected, deficiency in T cells reversed the inhibition of tumor growth observed in the Ifi35^ko^ group (Fig. 3B, Supplementary Fig. S8A). Furthermore, in vivo depletion studies showed that tumor growth in the Ifi35^ko^ group was no longer inhibited when CD8^+^T cells were depleted (Fig. 3C, Supplementary Fig. S8B). This suggested that CD8+ T cells are required for Ifi35^ko^ tumors. Collectively, these results indicate that tumor-intrinsic IFI35 drives the infiltration of MDSCs, preventing the actions of effector CD8^+^ T cells and ultimately accelerating TNBC tumor progression.

### IFI35 orchestrates an immunosuppressive microenvironment in TNBC via CCL2

**Fig. 4:**
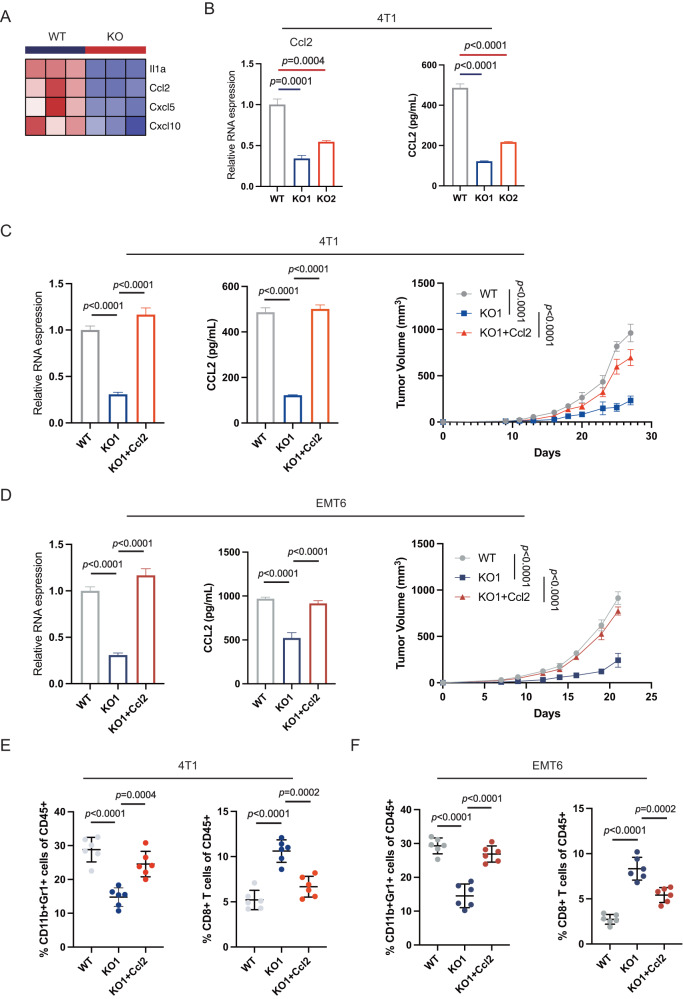
IFI35 orchestrates tumor immune microenvironment by CCL2. **A** Heatmap showing log2 fold change in Il1α, Ccl2, Cxcl5, Cxcl10 expression in Ifi35^ko^ or WT 4T1 cell lines (*n* = 3 per group). **B** RT-qPCR and ELISA analysis for CCL2 from Ifi35^ko^ or WT 4T1 cell lines (*n* = 3) (one-way ANOVA test with Turkey’s multiple comparisons, mean with SD is plotted). **C**, **D**. RT-qPCR analysis, ELISA analysis, and tumor growth curves of Ifi35^ko^, Ifi35^ko^ +Ccl2, and WT 4T1 (**C**) and EMT6 (**D**) cell lines (*n* = 3) (one-way or two-way ANOVA test with Turkey’s multiple comparisons, mean with SD is plotted). The frequencies of MDSCs and CD8^+^T cells population in balb/c mice inoculated with Ifi35^ko^, Ifi35^ko^ +Ccl2, and WT 4T1 (**E**) or EMT6 (**F**) cell lines (*n* = 6) (one-way ANOVA test with Turkey’s multiple comparisons, mean with SD is plotted).

To explore how IFI35 induces an immunosuppressive microenvironment in TNBC, we performed RNA-seq with the Ifi35^ko^ and WT 4T1 tumor cells. Significant changes in cytokine-related pathways have been found in Gene Set Enrichment Analysis (Supplementary Fig. 9). Considering that IFI35 promotes the infiltration of MDSCs and ultimately leads to the anergy of CD8^+^T cells, we focused on cytokines with chemotactic effects. Il1α, Ccl2, Cxcl5, and Cxcl10 were significantly downregulated in Ifi35^ko^ 4T1 tumor cells (Fig. 4A, Supplementary Table 2). Then, we examined the mRNA expression of these 4 genes in Ifi35^ko^ and WT 4T1 or EMT6 tumor cells using RT-qPCR. Ccl2 was the most obviously downregulated of the four DEGs (Fig. 4B, Supplementary Fig. 10A–D). Previous studies have demonstrated that CCL2 can recruit MDSCs infiltration to form an immunosuppressive microenvironment, which helps tumor cells evade the attack of immune cells and supports tumor cell proliferation [26]. Since CCL2, as a secreted factor, plays a role in immune cell chemotaxis through the CCR2 receptor, we extracted the supernatants of cultured tumor cells for ELISA. Compared with that in the supernatant from WT tumor cells, the level of secreted CCL2 in the supernatant from Ifi35^ko^ 4T1 or EMT6 tumor cells was significantly reduced (Fig. 4B, Supplementary Fig. S10D).

To confirm the mechanism by which IFI35 reshapes the TNBC immune microenvironment, the Ifi35^ko^ 4T1 or EMT6 TNBC tumor cells were engineered to overexpress Ccl2. Both RT-qPCR and ELISA confirmed that Ifi35^ko^ 4T1 and EMT6 tumor cells overexpressing Ccl2 had increased mRNA expression and secretion of CCL2 (Fig. 4C, D). In addition, the inhibition of the growth of Ifi35^ko^ tumors was reversed by overexpressing of Ccl2 in Ifi35^ko^ 4T1 or EMT6 tumors (Fig. 4C, D). In line with this phenomenon, flow cytometry analysis found that Ifi35^ko^ tumors overexpressing Ccl2 had increased MDSCs infiltration and decreased CD8^+^T cells infiltration (Fig. 4E, F). Taken together, our results show that IFI35 promotes the secretion of CCL2, which leads to the infiltration of MDSCs to form an immunosuppressive microenvironment. As a result, the antitumor effect of CD8^+^ T cells is inhibited, leading to TNBC tumor progression.

### IFI35 ablation enhances the efficacy of immunotherapy

**Fig. 5:**
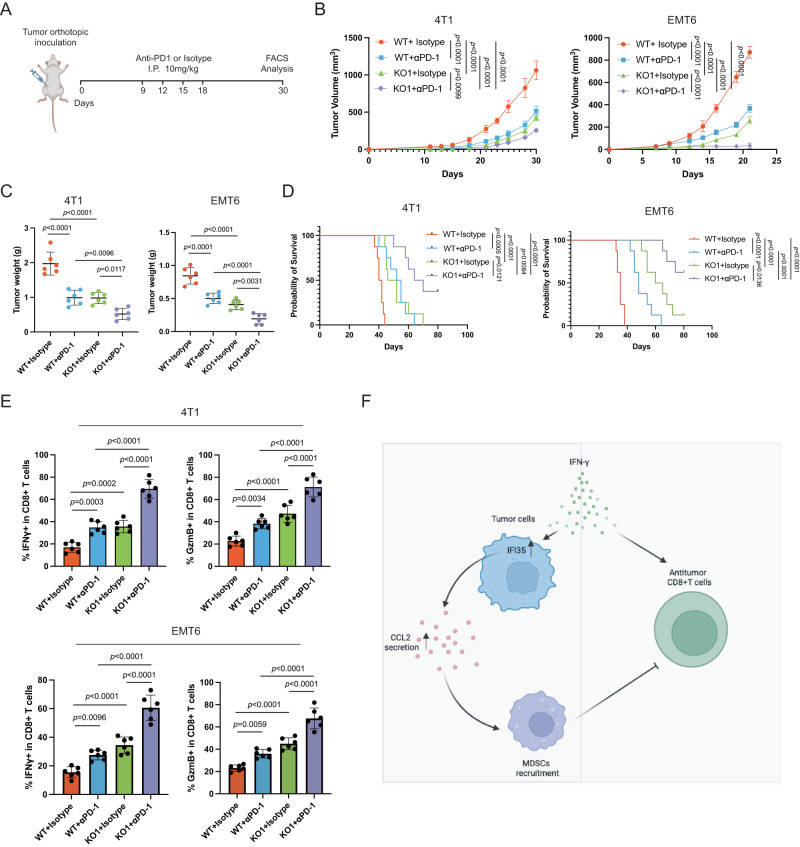
IFI35 ablation combined with PD-1 blockade synergistically inhibits TNBC progression. **A** Schematic of tumor inoculation and antibody injection in mice. **B** Tumor growth curves of indicated 4 groups (WT + Isotype, WT + αPD-1, Ifi35^ko^ + Isotype, Ifi35^ko^ + αPD-1) (*n* = 6 per group, two-way ANOVA test, mean with SD is plotted). **C** Tumor weight 30 days after tumor inoculation in indicated 4 groups (WT+Isotype, WT + αPD-1, Ifi35^ko^ + Isotype, Ifi35^ko^ + αPD-1) (*n* = 6 per group, one-way ANOVA test with Turkey’s multiple comparisons, Mean with SD is plotted). **D** Kaplan–Meier curves for the survival of indicated 4 groups of balb/c mice (WT + Isotype, WT + αPD-1, Ifi35^ko^ + Isotype, Ifi35^ko^ + αPD-1) (*n* = 8 per group, log-rank test). **E** The frequencies of IFNγ^+^CD8^+^ and GzmB^+^CD8^+^ T cells population in indicated 4 groups of balb/c mice (WT + Isotype, WT + αPD-1, Ifi35^ko^ + Isotype, Ifi35^ko^ + αPD-1) (*n* = 6 per group, one-way ANOVA test with Turkey’s multiple comparisons, mean with SD is plotted). **F** Model illustrating how IFI35 orchestrates tumor microenvironment to promote TNBC progression.

Although the PD-1/PD-L1 ICIs have been approved for the treatment of advanced and metastatic TNBC, few patients obtained durable clinical responses [3]. Also, we explored whether IFI35 or CCL2 affect PD-L1 expression in tumor cells. As a result, it showed that neither IFI35 ablation in WT tumor cells nor CCL2 rescue in Ifi35^ko^ tumor cells affected the expression of PD-L1 (Supplementary Fig. 11A, B). Given that the increased CD8^+^T cells infiltration in the Ifi35^ko^ tumors, we hypothesized that IFI35 ablation would sensitize TNBC tumors to immunotherapy. Nine days after Ifi35^ko^ and WT 4T1 or EMT6 tumors inoculation, mice were treated with anti-PD-1 immunotherapy (Fig. 5A). We observed that abrogation of IFI35 or anti-PD-1 treatment alone inhibited tumor growth in and prolonged overall survival of mice (Fig. 5B–D). However, combination therapy with IFI35 ablation and anti-PD-1 treatment showed a significant reduction in tumor growth and obviously prolonged survival (Fig. 5B–D). Subsequently, flow cytometry analysis further confirmed that the combined treatment significantly increased the proportions of IFNγ^+^CD8^+^T and GzmB^+^CD8^+^T cells (Fig. 5E). Taken together, our results confirm that the IFI35 ablation and anti-PD-1 combination therapy obviously inhibited tumor growth and extended survival, suggesting a synergistic therapeutic effect.

## Discussion

Several classic clinical experiments have indicated that PD-1/PD-L1 ICIs can increase the objective response rate of TNBC patients and prolong patient survival [27–30]. These findings have established a position of PD-1/PD-L1 immunotherapy as auxiliary and neoadjuvant therapy for TNBC. Despite these successes, a variety of resistance mechanisms still exist in TME and hinder the efficacy. Specifically, antigen loss, the infiltration of immunosuppressive immune cells, the activation of immune inhibitory receptors are all main causes of T cell dysfunction [31]. To overcome these obstacles, we previously identified a new key gene, IFI35, that participates in immune regulation in TNBC. In this study, we further revealed that IFI35 can be induced by IFN-γ in the TME. In addition, IFI35 did not exert a crucial role in immune cells but rather promoted the progression of TNBC in a tumor-intrinsic manner.

IFN-γ is a pleiotropic cytokine that plays a crucial role in the TIME [5]. On the one hand, IFN-γ can induce cell cycle arrest [32], apoptosis [33], and ferroptosis [34] through the intrinsic regulatory mechanisms to exert anti-tumor effects. Furthermore, IFN-γ also promotes the processing and presentation of tumor cell antigens, thereby improving immunogenicity and affecting the cleavage of exogenous polypeptides in the immune system [35]. Through an extrinsic mechanism, IFN-γ upregulates the expression of MHC molecules and co-stimulatory molecules, induces CD4^+^ T cells and macrophages to differentiate into Th1 and M1 subtypes, respectively, and ultimately promotes the cytotoxicity of CD8^+^ T cells and tumor cell cytolysis mediated by NK cells [36]. On the other hand, long-term exposure to the sustained IFN-γ stimulation can induce the upregulation of immunosuppressive metabolites such as arginine and IDO in the microenvironment and induce the expression of downstream PD-L1, which in turn mediates the immune escape of tumor cells [37, 38]. It can be seen that IFN-γ is a double-edged sword in tumors, not only enhancing immunity against tumors but also inducing immune suppression and promoting tumor progression. As an interferon-stimulated gene (ISG), tumor-derived IFI35 was confirmed by us to promote MDSCs infiltration, and eventually lead to the dysfunction of CD8^+^T cells in TNBC. In summary, IFI35 exerts a negative immune negative regulatory role similar to that of PD-L1 in the TIME.

Previous studies have indicated that tumor cells can produce CCL2, which induces the infiltration and migration of various immune cells and plays a crucial role in the immune response [39]. For example, activation of the CCL2-CCR2 axis promotes the migration and differentiation of M2 macrophages, which drives the formation of an immunosuppressive microenvironment, ultimately leading to ovarian cancer progression [40]. Another report showed that CCL2 induced MDSCs migration to support glioma development [41]. In our study, we found that IFI35 promoted the secretion of tumor-derived CCL2, which led to the infiltration of MDSCs to promote TNBC immune escape.

In our previous study, higher IFI35 expression tended to be associated with a better prognosis [21]. However, IFI35 was identified as an oncogene in this study, which seems to be contradictory. We give the following explanation for this. IFN-γ is a potent antitumor cytokine that promotes the formation of an inflammatory antitumor immune microenvironment in TNBC. IFI35 is a sensitive IFN-stimulated gene, and its expression reflects the level of secreted IFN-γ in the environment. Naturally, high IFI35 expression thus is a marker of good prognosis. However, sustained inflammatory stimuli also induce the expression of immunosuppressive molecules such as PD-L1, thereby preventing normal cells from being damaged by excessive immune responses [2, 42]. Similar to PD-L1, the mechanisms of IFI35 are hijacked by tumor cells, with IFI35 ultimately accelerating TNBC progression.

In summary, IFN-γ is a double-edged sword. In TNBC, it not only promotes the antitumor immune killing of CD8^+^ T cells but also upregulates IFI35 expression (Fig. 5F). IFI35 further promotes CCL2 secretion, which eventually leads to the infiltration of MDSCs and the dysfunction of antitumor CD8^+^ T cells. IFI35 ablation improves responsiveness to anti-PD-1 therapy, and it is expected to become a new immunotherapy target.

## Methods

### Mice

Ifi35^−/+^ mice were generated by Shanghai Model Organisms using a CRISPR/cas9-mediated genome engineering strategy. Ifi35^−/−^ mice were generated from an initial heterozygous breeding (details in Supplementary Fig. S3). Balb/c and nude mice were obtained from Charles River. All mice were housed at Fudan University Shanghai Cancer Hospital Experimental Animal Center under pathogen-free conditions and in individually ventilated cages. Experimental mice were female and aged 6–8 weeks. All animal experiments were approved by the Ethics Committee of Fudan University Shanghai Cancer Hospital (FUSCC-IACUC-2022044, IACUC-2022096).

### Cell lines

The HEK-293T (CRL-3216), BT-549 (HTB-122), MDA-MB-231 (CRM-HTB-26), EMT-6 (CRL-2755) and 4T1 (CRL-2539) were purchased from the American Type Culture Collection (ATCC). All cell lines were cultured in appropriate culture media supplemented with 1% Penicillin/Streptomycin and 10% fetal bovine serum (FBS; Gibco), and maintained at 37 °C and 5% CO_2_. Cell lines have been STR-authenticated and were routinely tested for mycoplasma-free.

4T1 and EMT6 Ifi35^ko^ cells were generated by CRISPR/cas9-mediated genome engineering. The CRISPOR tool was used to design the targeting sequence and sequence information were: sgRNA_1 (5’- CAC-CGT-TTG-GCC-TTG-GAA-TAC-CAG-3’), sgRNA_2 (5’-CAC-CGA-TTC-CAA-GGC-CAA-ACT-AAGC-3’), sg_Control (5’-CAC-CGT-TCG-GCT-GGT-GTG-CGT-TCAC-3’). These CRISPR guide RNAs were cloned into the LentiCRISPR v2 plasmid (Addgene, 52961) using the BsmBI site. The cloned LentiCRISPR vectors containing the sgRNAs were transfected into 293T cells along with both psPAX2 and pMD2.G plasmids simultaneously using PEI Transfection (Teyebio). Virus-containing supernatants were harvested and filtered through a 0.45 μM filter. Supernatant and polybrene (4 μg/ml) were used to infect cells. Then, cells were subjected to puromycin (2 μg/ml) selection for 48 h, and off-target effects were determined by immunoblotting.

To generate Ccl2 overexpressing 4T1 or EMT-6 Ifi35^ko^ cell line, the whole coding sequence of Ccl2 gene was cloned into pLVX-IRES-Neo (Takara, 632181) vetor using EcoRI and XbaI sites. Retroviral production and cell infection were performed as previously described. Then, cells were selected for 7 days with G418 disulfate (500 μg/ml, Sigma). Overexpression was confirmed by RT-qPCR and ELISA.

### Tumor model

4T1 and EMT6 cells in exponential growth phase were digested with trypsin, washed three times with cold PBS. After centrifugation, cells were resuspended in cold PBS. 100 μl cold PBS containing 1 ×10^5^ (4T1) or 5 ×10^5^ (EMT6) cells was orthotopically inoculated on the right flank of the fourth pair of mammary fat pads of mice. For PD-1 blockade experiment, 10 mg/kg anti-PD-1 (BE0146, BioXCell) or Isotype control (BE0089) were given by intraperitoneally (i.p.) every 3 days for a total of 4 times starting on day 9 after tumor inoculation. Tumor growth was monitored with a caliper and volume was calculated as (length × width^2^)/2. All tumor volumes did not exceed 1500 mm^3^.

### Quantitative RT-qPCR

Total RNA was extracted by using the traditional Trizol-chloroform-isopropanol method. RNA was quantified using a Nanodrop instrument. cDNA synthesis, RT-qPCR process, and data analysis are the same as our previous description [21]. All primer sequences can be found in Supplementary Table 3.

### Western blotting

Cultured cells were lysed with RIPA cell lysis and extraction buffer (ThermoFisher) supplemented with Halt^TM^ Protease and Phosphatase Inhibitor Cocktail (ThermoFisher). Protein concentrations were measured using BCA Protein Assay kit (Solarbio). Protein concentrations were normalized with 5× Native Gel Sample Loading Buffer (NCM biotech) and heated at 100 °C for 10 min. Proteins were separated electrophoretically on 10–15% polyacrylamide gel (Bio-Rad) and transferred to PVDF membrane Trans-Blot Turbo transfer system (Bio-Rad). Before incubating with primary antibodies, the membranes were blocked with PBS containing 5% non-fat milk powder and 0.1% Tween. Then, the membranes were incubated with primary antibodies against IFI35 (1:1000, Abcam, #233415; 1:100, Santa Cruz, #393513), β-Actin (1:5000, CST, #3700S) overnight at 4 °C. After washing with PBST, the membranes were incubated with appropriate HRP-linked secondary antibodies (Anti-Rabbit and Anti-Mouse; Abcam) for 1 h at room temperature. Membranes were washed again and incubated with Pierce ECL Western Blotting Substrate (ThermoFisher) and imaged using ChemiDoc^TM^ XRS+ System (Bio-Rad).

### Immunohistochemistry

4μm thick formalin-fixed, paraffin-embedded tissue sections were used to perform immunohistochemical staining. After deparaffinization and rehydration process, sections were heated in a pressure cooker in EDTA or citrate buffer for antigen retrieval, and then were blocked by peroxidase endogenous blocking solution (Vector) for 15 min and protein block (Abcam) for 1 h. Sections were incubated with primary antibodies against IFI35 (1:100, Abcam, #233415) overnight at 4 °C in a humid chamber. After washing with PBS, sections were incubated with HRP-conjugated Goat Anti-Rabbit/Mouse secondary antibody (Genetech) at room temperature for 30 min in a humid chamber, followed with 3,3′- diaminobenzidine staining for 3–5 min under the microscope. Nuclei were counterstained with hematoxylin. Finally, sections were dehydrated in a series of alcohols and mounted in neutral gum. Two investigators who had never seen the clinical record semi-quantitatively scored the slides by evaluating percentage of stained cells and the staining intensity in representative areas. The percentage of cells stained was scored on a scale of 1–4 (1, 1–25%; 2, 26–50%; 3, 51–75%; 4, 76–100%). The staining intensity was scored on a scale of 1–3 (1, weak; 2, intermediate; 3, strong). IFI35 expression of each sample was evaluated by computing a final immunohistochemistry score as the multiplication of the percentage and intensity score. The sample score of 0–12 was obtained. Representative sections were scanned by Pannoramic DESK system (3DHISTECH).

### Flow cytometry

Tissues were collected in ice-cold RPMI-1640 supplemented with 1% Penicillin/Streptomycin and 10% fetal bovine serum (FBS; Gibco). Tumor samples were minced into small fragments and digested with Collagenase I (0.5 mg/ml, Worthington), Hyaluronidase (1 mg/ml, Sigma) and DNase I (0.01 mg/ml, Roche) in gentleMACS^TM^ Dissociator (Miltenyi). Cell suspenions or spleen samples were filtered with 70 μm cell strainer (Biologix). Single-cell suspensions were treated with RBC lysis buffer (Solarbio) to remove red blood cells. Then, single-cell suspensions were resuspended and counted 1 ×10^6^ cells in PBS. Fixable Viability Stain 780 (1:1000, BD Biosciences) was added to exclude dead cells. Then, cells were treated with purified Rat Anti-Mouse CD16/CD32 antibody (BD Biosciences) for 15 min, and subsequently incubated with different combinations of fluorescently labeled monoclonal antibodies against CD45, CD3, CD4, CD8, CD25, NKp46, PD-1, CD11b, CD11c, Gr1, Ly6C, Ly6G, F4/80, CD86, and CD206 for 30 min in the dark at 4 °C. For intracellular cytokine staining of TILs, cells were stimulated in vitro with Leukocyte Activation Cocktail (BD Biosciences) for 4 h, and surface stained as previously described. Then, cells were fixed and permeabilized using Transcription Factor Buffer Set (BD Biosciences) for 50 min and stained with antibodies against IFN-γ, GzmB, and Ki-67 for 50 min. Sample analysis were performed on Beckman Coulter CytoFlex S, and data were analyzed by FlowJo software.

### RNA sequencing

Total RNA was extracted using Trizol reagent kit (Invitrogen). RNA quality was assessed on an Agilent 2100 Bioanalyzer (Agilent Technologies) and checked using RNase free agarose gel electrophoresis. After total RNA was extracted, eukaryotic mRNA was enriched by Oligo(dT) beads. Then the enriched mRNA was fragmented and reversely transcribed into cDNA by using NEBNext Ultra RNA Library Prep Kit for Illumina(NEB).The purified double-stranded cDNA fragments were end repaired, and ligated to Illumina sequencing adapters. The ligation reaction was purified with the AMPure XP Beads(1.0X). The resulting cDNA library was sequenced using Illumina Novaseq6000 by Gene Denovo Biotechnology Co. (Guangzhou, China). Quality control and data filter of the raw sequences were performed by fastq. Reads were aligned to the mouse reference genome (Mus musculus, GRCm39) using HISAT2. The RSEM software was used to quantify gene expression abundance. Differential expression analysis was performed by DESeq2. Genes with absolute fold change >2 and *p* value < 0.05 were considered as differentially expressed genes. Gene sets enrichment analysis was performed using GSEA software.

### ELISA

To analyze cytokines secreted by tumor cells, cells were plated at same density and ensure the same volume of culture medium. After 48 h of cell culture, cell supernatants were extracted. The concentration of CCL2 (Absin) was detected using Enzyme linked immunosorbent assay according to manufacturer’s protocol.

### Statistical analysis

GraphPad prism v9.4.1 was used for all statistical analysis. In the correlation analysis, the pearson test was applied. For the comparison of more than two groups, one-way or two-way ANOVA test with Turkey’s multiple comparisons was performed. Log-rank test was applied for survival analysis. *p* valu*e* < 0.05 was considered as significant.

### Supplementary information

Supplementary materials

Supplementary Table 1

Supplementary Table 2

Supplementary Table 3

## Data Availability

All fastq files of RNA-seq data were uploaded to SRA datasets with accession number SRP427359.
