# Supplementary material for: IFI35 limits antitumor immunity in triple-negative breast cancer via CCL2 secretion
Source: Oncogene. 2024 Jan 12;43(10):693–702. doi: 10.1038/s41388-023-02934-w (PMC10907302; doi:10.1038/s41388-023-02934-w)
Supplement: Supplementary file 1 — Supplementary materials [file 41388_2023_2934_MOESM1_ESM.pdf]

IFI35 limits antitumor immunity in triple-negative breast cancer via the pathway of CCL2 secretion

Baojin Xu[1,2,3], Hefen Sun[1,2*], Simeng Liu[1,2], Li Liao[1,2], Xiaoqing Song[1,2], Yi Wu[1,2], Yifeng Hou[1,2,] and Wei Jin[1,2*]

1. Key Laboratory of Breast Cancer in Shanghai, Fudan University Shanghai Cancer Center, Shanghai 200032, China
2. Department of Oncology, Shanghai Medical College, Fudan University, Shanghai 200032, China
3. Department of Breast Surgery, Liaoning Cancer Hospital and Institute, Cancer Hospital of China Medical University, Shenyang 110042, China

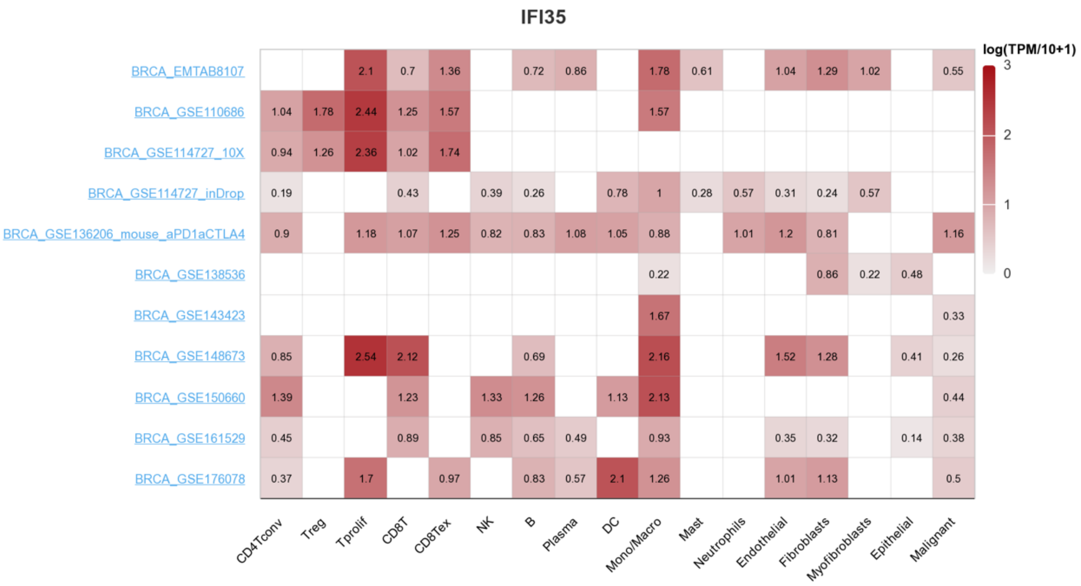

Supplementary Figure 1. IFI35 expression in TNBC tumor microenvironment in the TISCH scRNA-seq database.

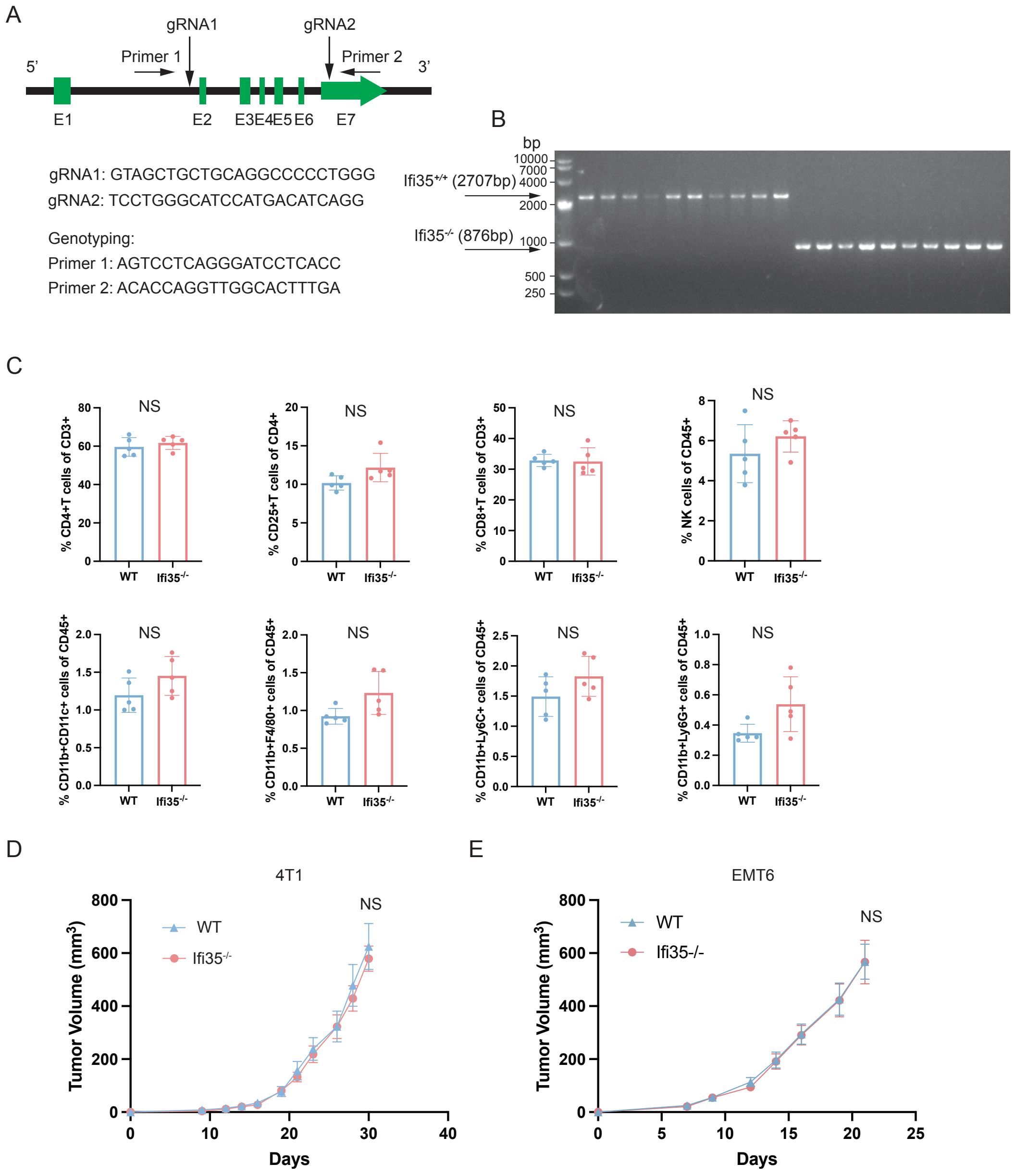

Supplementary Figure 2. Ifi35$^{-/-}$ mice do not affect antitumor immune response. A. Cartoon of the strategy to generate Ifi35$^{-/-}$ mice with a CRISPR/Cas9-mediated genome engineering strategy. The sequences of two guide RNA and the primers used for genotyping were shown. B. Genotyping results for WT or Ifi35$^{-/-}$ alleles. C. Flow cytometry analysis of CD4+T cells, regulatory T cells (CD4+CD25+), CD8+T cells, natural killer cells (NKp46+), conventional dendritic cells (CD11b+CD11c+), macrophage (CD11b+F4/80+), monocytes (CD11b+Ly6C+), and neutrophils (CD11b+Ly6G+) in spleen from wild-type and Ifi35$^{-/-}$ mice (n = 5, two tailed Student's t-test, mean with SD). D. The tumor growth curve of wild-type and Ifi35$^{-/-}$ mice inoculated mammary fat pad with 4T1 or EMT6 TNBC cell line (n = 8-10, two-way ANOVA test, mean with SEM).

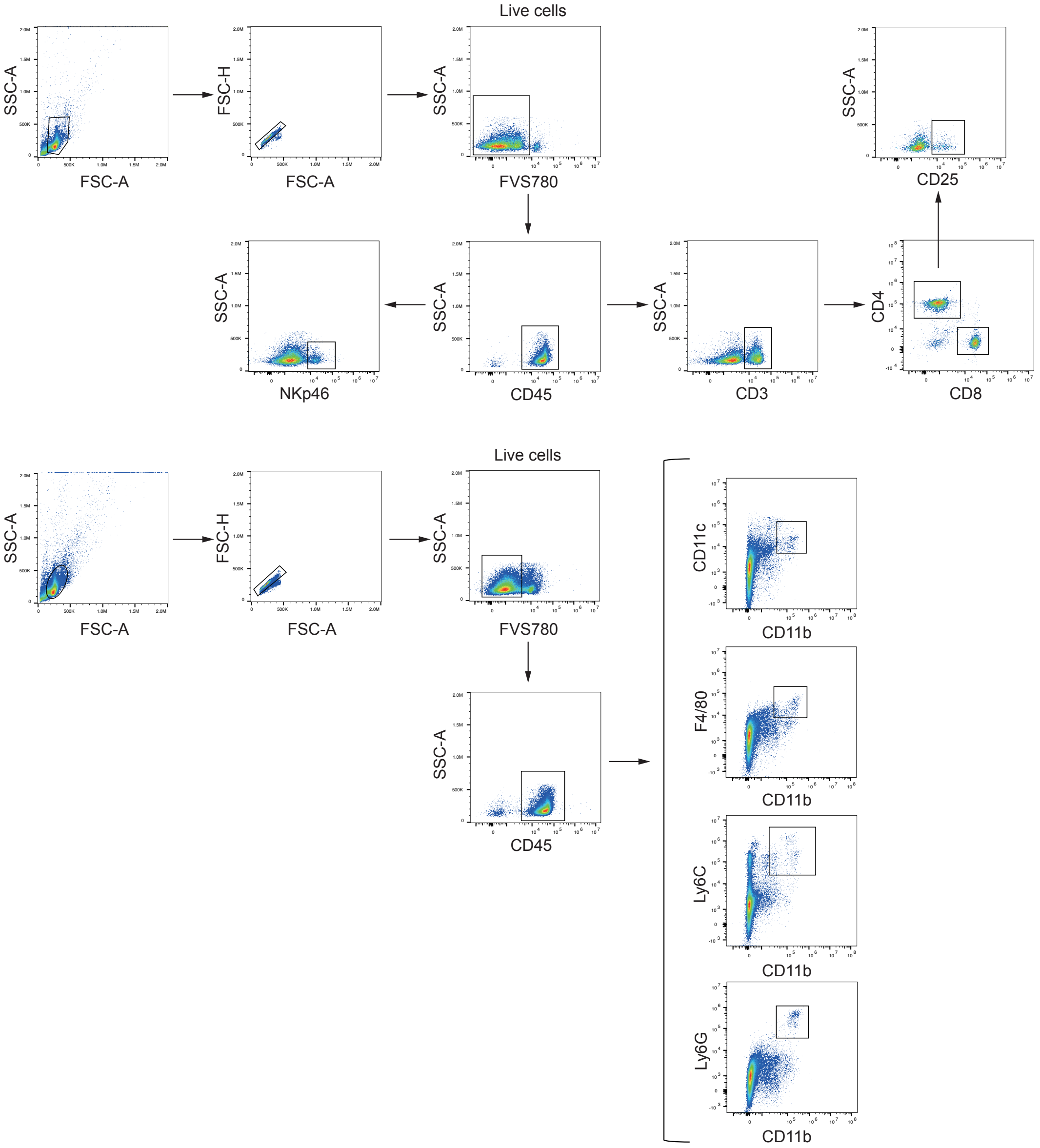

Supplementary Figure 3. Representative immune cell flow cytometry gating strategies of spleen analysis.

4T1

WT

Ifi35$^{-/-}$

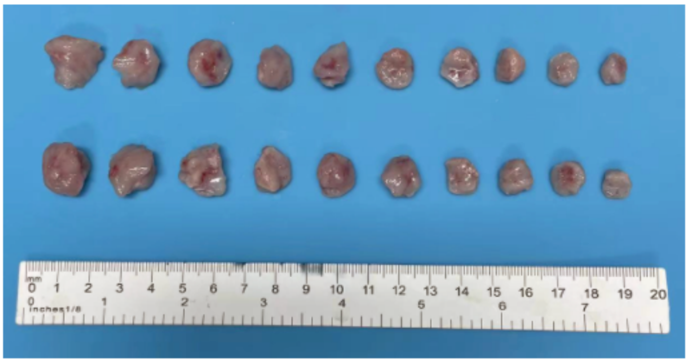

EMT6

WT

Ifi35$^{-/-}$

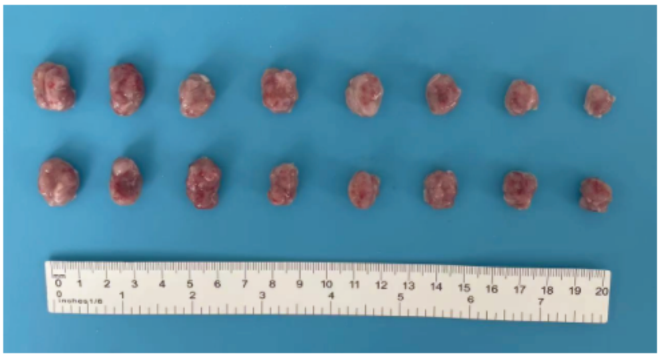

Supplementary Figure 4. Anatomical images of tumors explanted from wild-type and Ifi35$^{-/-}$ mice inoculated with wild type 4T1 or EMT6 tumors.

A

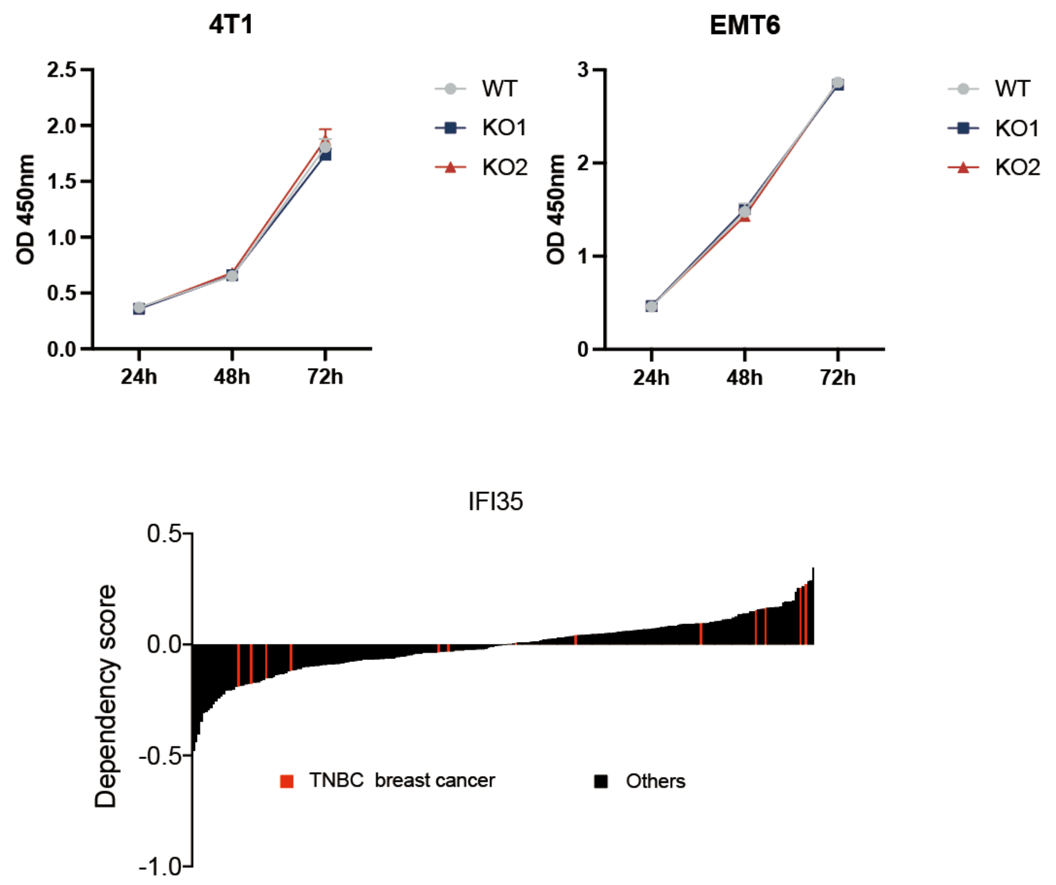

Supplementary Figure 5. IFI35 is independent in TNBC cell lines.

A. CRISPR-Cas9-based competitive proliferation assay against non-targeting sgRNA control cells in the 4T1 or EMT6 TNBC cell line (n = 3, two-way ANOVA test, mean with SD).

B. IFI35 dependency (CERES score) across 249 cell lines in the DepMap data set.

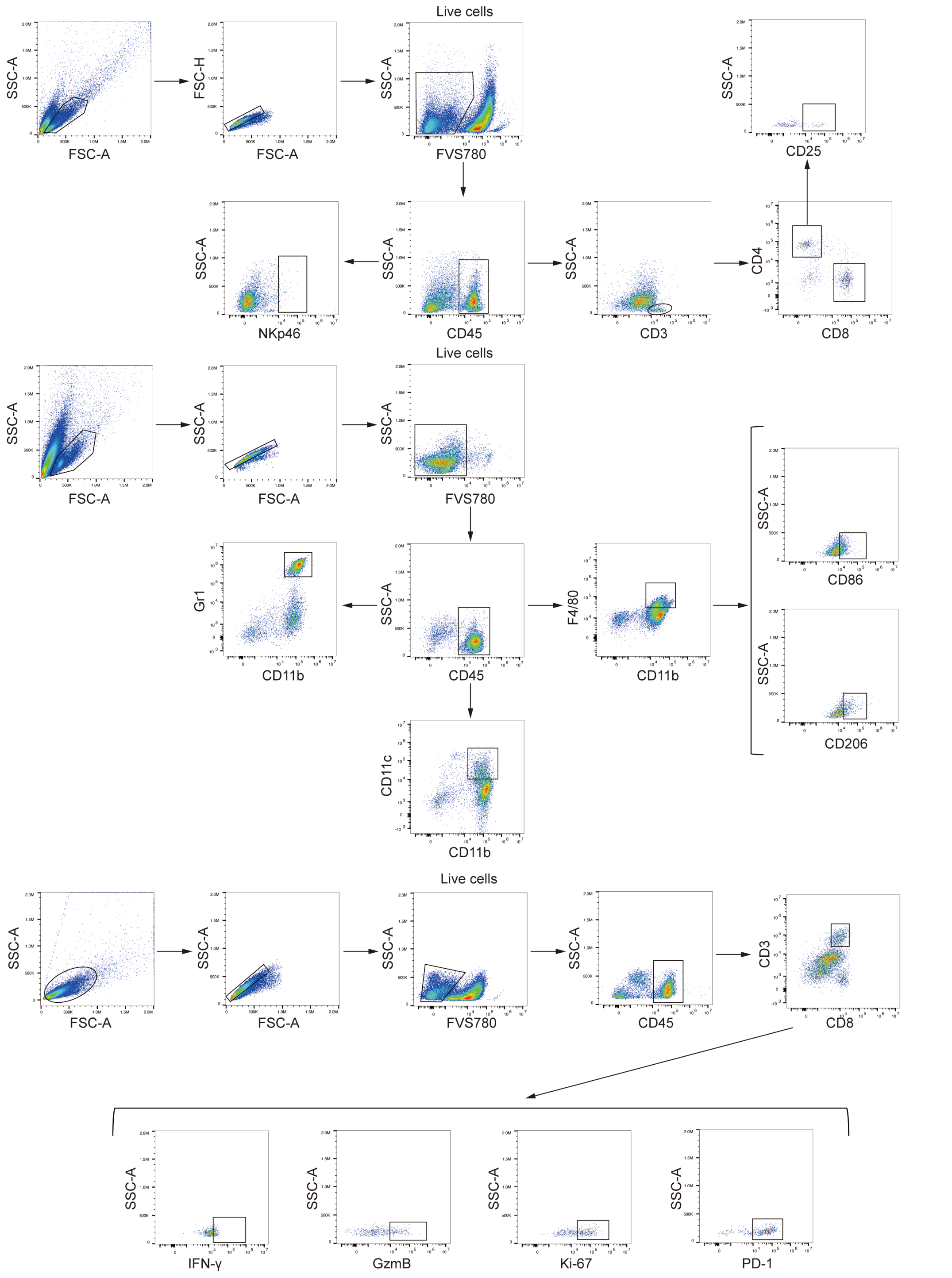

Supplementary Figure 6. Representative immune cell flow cytometry gating strategies of tumor infiltrating immune cell.

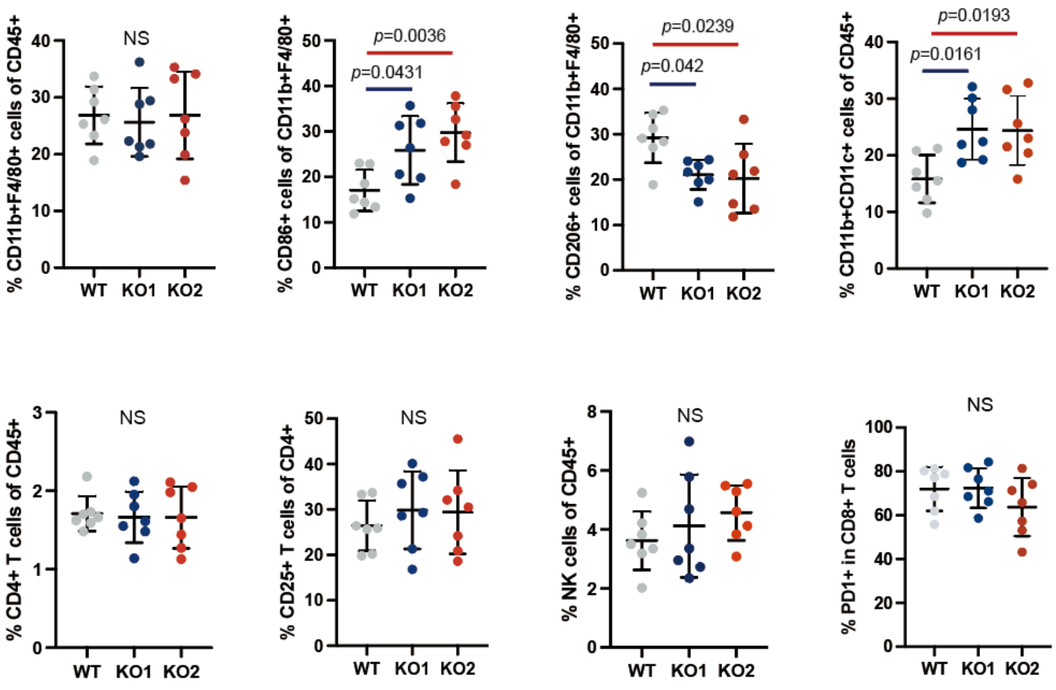

Supplementary Figure 7. IFI35 orchestrates tumor immune microenvironment. The frequencies of CD11b$^+$F4/80$^+$ macrophages, CD86$^+$ M1 macrophages, CD206$^+$ M2 macrophages, CD11b$^+$CD11c$^+$ DCs, CD4$^+$T cells, CD4$^+$CD25$^+$ T_{reg} cells, NKp46$^+$ NK cells, and PD1$^+$CD8$^+$T cells population in balb/c mice inoculated with Ifi35^{ko} or WT 4T1 tumor cells (n = 7, one way ANOVA test with Turkey's multiple comparisons, mean with SD is plotted).

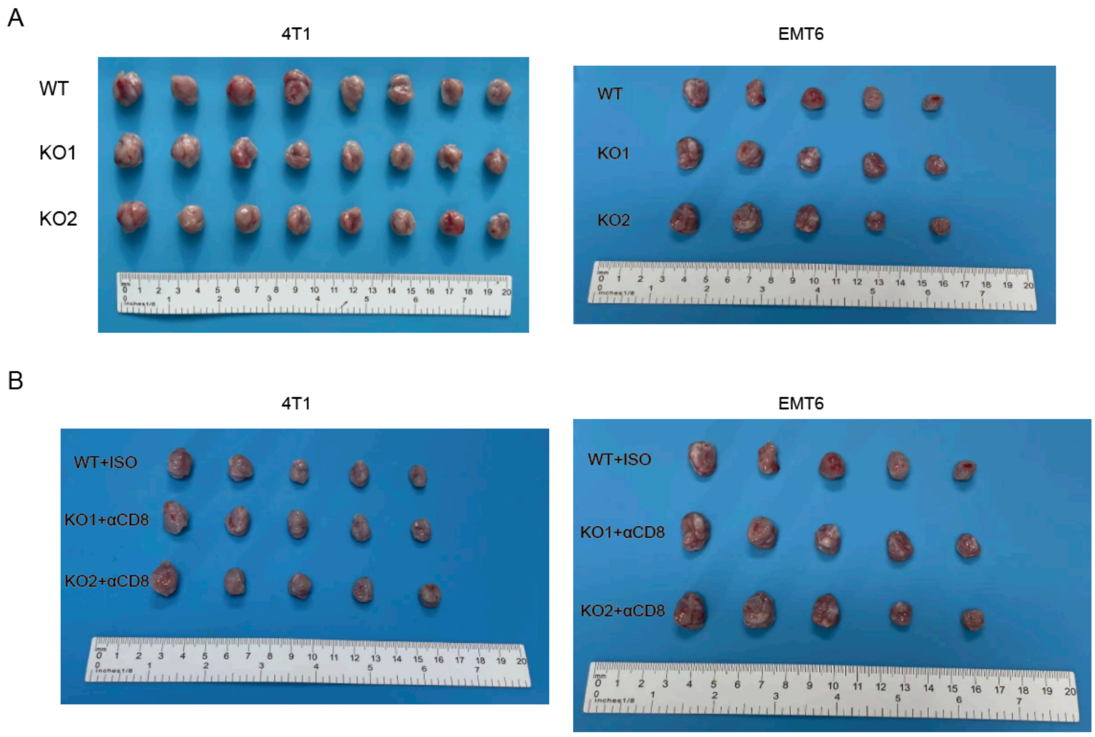

Supplementary Figure 8. A. Anatomical images of tumors explanted from nude mice inoculated mammary fat pad with Ifi35^{ko} or WT 4T1 and EMT6 tumor cells. B. Anatomical images of tumors explanted from Balb/c mice treated with anti-mouse CD8 antibody or isotype control.

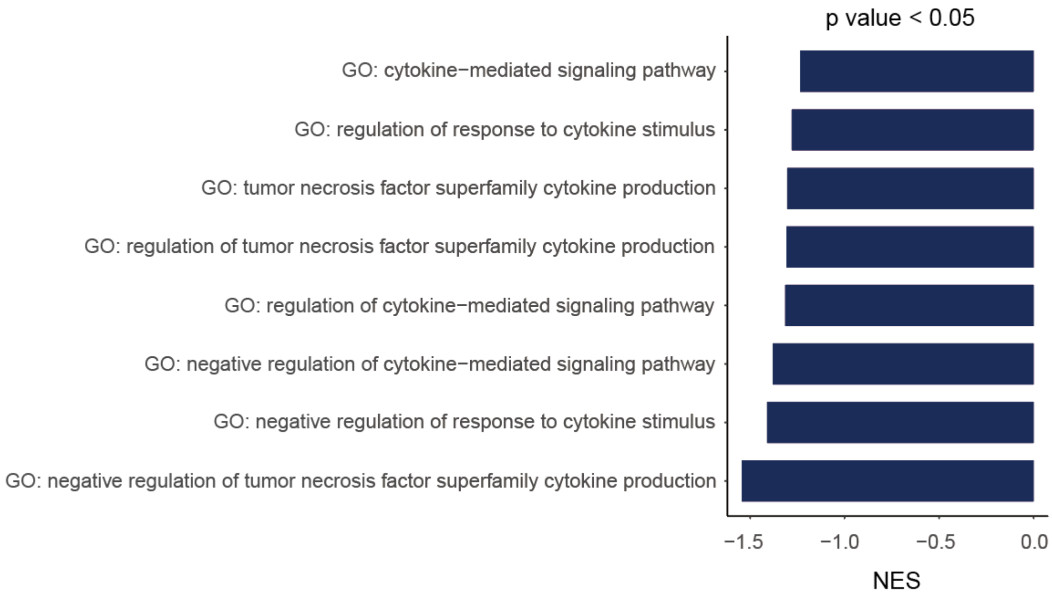

Supplementary Figure 9. Gene set enrichment analysis of WT and IFI35^{KO} 4T1 tumor cells. Significant changes in cytokine-related pathways were shown.

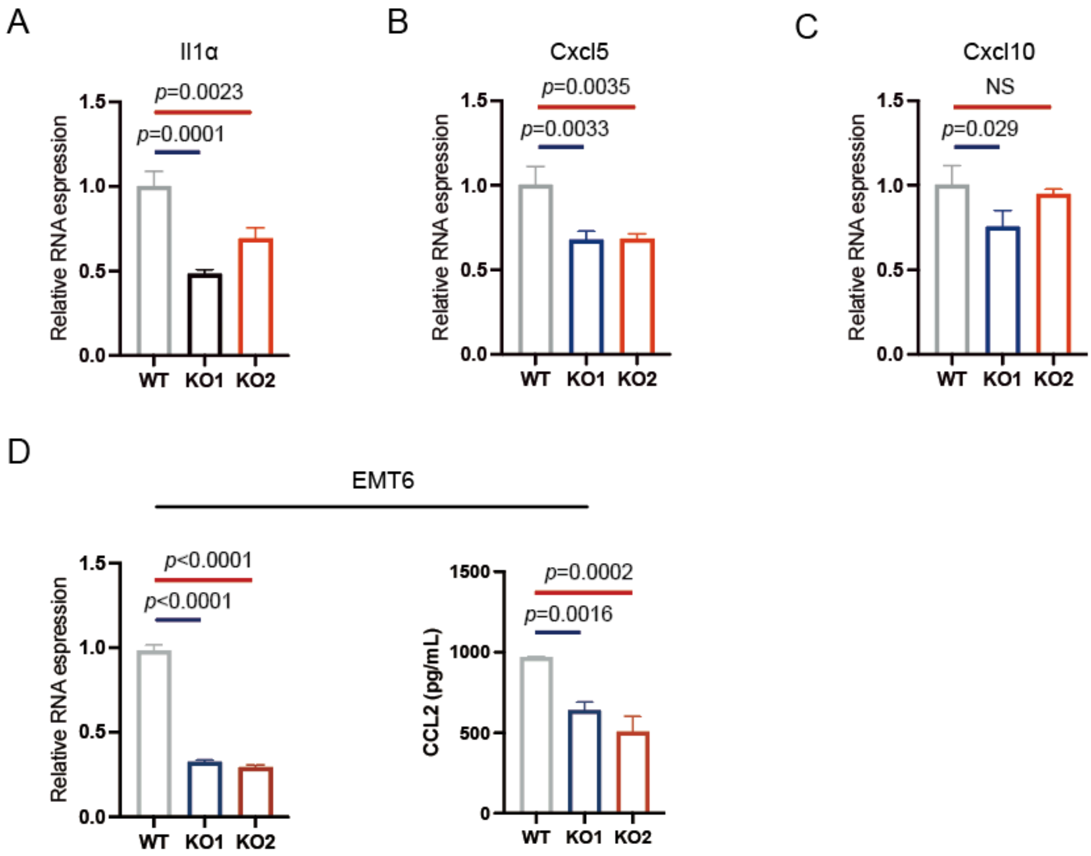

Supplementary Figure 10. IFI35 induces changes in cytokine production in TNBC. A-C. RT-qPCR analysis of Il1α (A), Cxcl5 (B) and Cxcl10 (C) mRNA expression in Ifi35^{ko} or WT 4T1 cell lines (n = 3) (one way ANOVA test with Turkey's multiple comparisons, mean with SD is plotted). D. RT-qPCR and Elisa analysis for Ccl2 from Ifi35^{ko} or WT EMT6 cell lines (n = 3) (one way ANOVA test with Turkey's multiple comparisons, mean with SD is plotted).

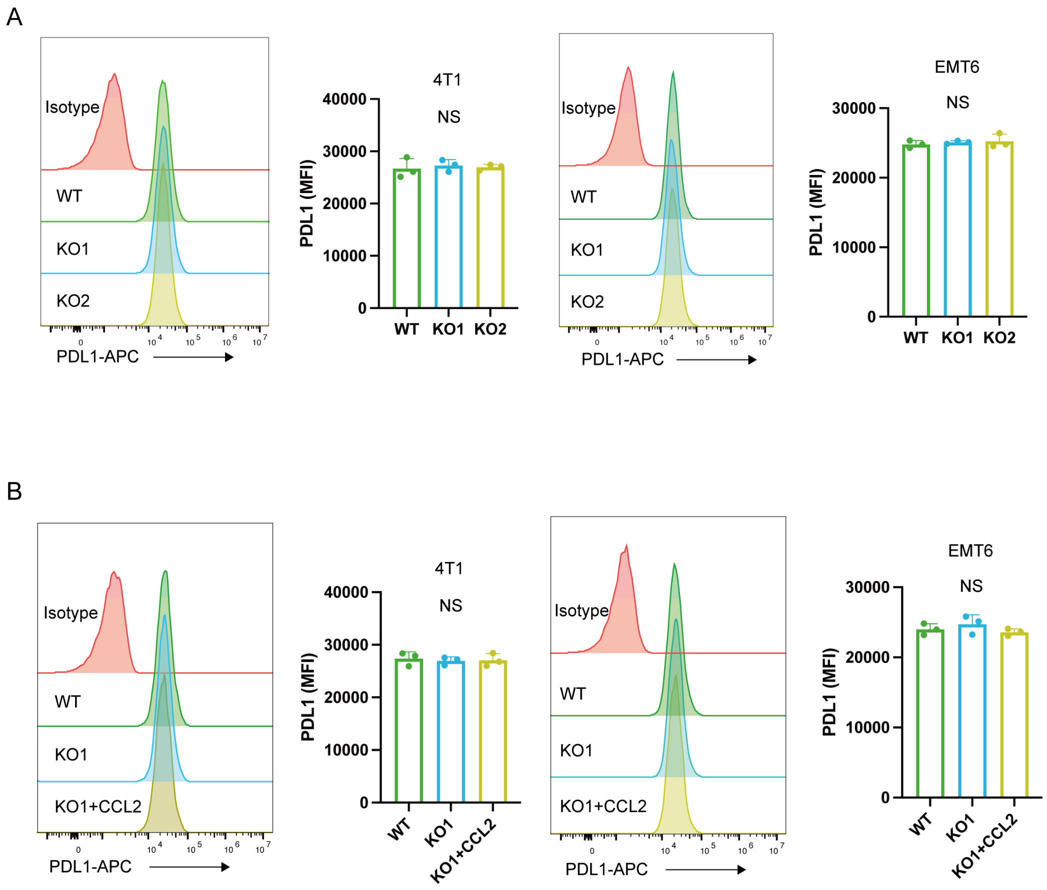

Supplementary Figure 11. IFI35 ablation or CCL2 rescue do not affect PDL1 expression in TNBC.

A-B. The analysis of PDL1 expression IFI35 ablation in WT tumor cells (A) or CCL2 rescue in Ifi35^{ko} tumor cells (B) (n = 3, one way ANOVA test with Turkey's multiple comparisons, mean with SD is plotted).
